# Can Chocolate Be Classified as an Ultra-Processed Food? A Short Review on Processing and Health Aspects to Help Answer This Question

**DOI:** 10.3390/foods12163070

**Published:** 2023-08-16

**Authors:** Cynthia Ditchfield, Marta Mitsui Kushida, Monica R. Mazalli, Paulo J. A. Sobral

**Affiliations:** 1 Department of Food Engineering, Faculty of Animal Science and Food Engineering, University of São Paulo, Pirassununga 13635-900, SP, Brazil; martakushida@usp.br (M.M.K.); mazalli@usp.br (M.R.M.); pjsobral@usp.br (P.J.A.S.); 2 Food Research Center (FoRC), University of São Paulo, Rua do Lago 250, Semi-Industrial Building, Block C, São Paulo 05508-080, SP, Brazil

**Keywords:** cocoa, fermentation, roasting, tempering, bioactive compounds, processed foods

## Abstract

Chocolate is a confectionery product whose consumption has increased, particularly dark chocolate. Chocolate is produced with varying amounts of cocoa liquor (CL), cocoa butter (CB) and cocoa powder (CP). The main chocolate types are dark, milk and white. Processing steps for chocolate production are described, and nutritional compositions examined for benefits and risks to health. Chocolate processing comprises steps at farm level, initial industrial processing for production of CL, CB and CP (common for all chocolate types) and mixing with other ingredients (like milk and sugar differing according to chocolate type) for industrial chocolate processing. All chocolate types present similar processing levels, and none involve chemical processing. Nutritional profiles of chocolate products differ according to composition, e.g., dark chocolate contains more CL, and so a higher antioxidant capacity. Chocolate is an energy-dense food rich in bioactive compounds (polyphenols, alkaloids, amino acids). Studies have demonstrated benefits of moderate consumption in reducing cardiovascular risk and oxidative and inflammatory burden, improving cognitive functions, maintaining diversity in gut microbiota, among others. In our view, chocolate should not be classified as an ultra-processed food because of simple processing steps, limited ingredients, and being an important part of a healthy diet when consumed in moderation.

## 1. Introduction

Chocolate is produced from a unique raw material: cocoa beans, but it can have other ingredients depending on the chocolate type. The history of cocoa is legendary in the American tropical regions, particularly in pre-Columbian Central America, where the Mayan civilization (250–900 AD) discovered that fermented and roasted cocoa beans had a special flavor and produced a beverage (“chocolatl”) used in religious rituals and as currency. Thus, Mayan peoples considered cocoa “the food of the gods” [1], and because of that, Carl Linnaeus named it *Theobroma cacao* L. in 1735. Recently, cocoa history was reviewed presenting evidence that it has been known since 3500 BC and originated in the Ecuadorian Amazonian region [2] and was taken to Europe by the Spanish where it became popular among royalty and was consumed with sugar to improve its bitter taste. The industrial evolution of cocoa processing into the popular confectionery product familiar to consumers nowadays was described by Silva et al. [3]. Readers interested in the history of cocoa/chocolate are invited to read the interesting review by Montagna et al. [1].

World production of cocoa beans reached almost 5.6 million tons in 2021, mainly by seven countries of three different continents: Ivory Coast (39.4% of world production), Ghana (14.7%), Indonesia (13%), Brazil (5.4%), Ecuador (5.4%), Cameroon (5.2%) and Nigeria (5%), according to Food and Agriculture Organization of the United Nations (FAO) data [4]. However, cocoa flavonoids impart a bitter taste that make cocoa beans revolting to humans in unprocessed form and so they must be processed, mainly into chocolate [5].

Food Engineering, Science and Technology (FEST) work to enhance food quality as with cocoa and chocolate. The search for high quality products with excellent sensory properties in the fine chocolate market has increased recently. For example, the chocolate market in Europe is constantly growing, mainly due to the increasing demand for dark chocolate for consumers who are interested in healthier, sustainable, and high-quality foods [6]. To this end, improvements in chocolate processing throughout the production chain from fermentation to packaging have been essential to achieving its desired flavor, texture and food functionality. As an example, much attention has been given to fermentation conditions that can improve chocolate sensory and nutritional properties by altering polyphenols, phytosterols, free fatty acids, fatty acid profile and volatile compounds, which yields products with higher nutritional value and with properties or containing ingredients that have a positive effect on human health [7,8,9].

Chocolate has been appreciated since ancient times for its medicinal properties, and as a dense source of high energy [2]. Now, it is still consumed worldwide for pleasure as well as health benefits; however, the relationship between chocolate and health in humans should be clearly established [1]. Strangely, chocolate has been considered an unhealthy food [10], certainly because it was classified as an ultra-processed food (UPF) [11]. Thus, the objectives of this short review are to describe and discuss chocolate’s processing from farm to industry to consumer, to illustrate there are no extreme treatments or chemical processes and so state that it cannot be considered a UPF, and to discuss its consumption risks and benefits by demonstrating that chocolate processing can contribute to a healthy diet.

## 2. Food Classification According to NOVA

A definition has been created so that some industrialized foods can be considered “ultra-processed foods” (UPF), in the context of a new classification (NOVA) that distributes foods into four groups [11]:

(1) Unprocessed and minimally processed foods (MPF);
(2) Processed culinary ingredients;
(3) Processed foods (PF);
(4) “ultra-processed” foods (UPF).

NOVA stated that UPF are formulations of ingredients, most of exclusive industrial use, typically created by a series of industrial techniques and processes (hence “ultra-processed”). According to this reference, some common UPF are carbonated soft drinks; sweet, fatty, or salty packaged snacks; candies (confectionery); chocolates; packaged bread and buns, cookies (biscuits), pastries, cakes and cake mixes; margarine and other spreads; sweetened breakfast “cereals” and fruit yogurt and “energy” drinks; pre-prepared meat, cheese, pasta and pizza dishes; poultry and fish “nuggets” and “sticks”; sausages, burgers, hot dogs and other reconstituted meat products; powdered and packaged “instant” soups, noodles and desserts; baby formula. NOVA stated that their consumption should be avoided [11].

According to Bonaccio et al. [10], UPF (including chocolate) are unhealthy and increase the risk for many chronic diseases and could increase the inflammatory potential of the diet, impacting immunity and lung function, thus increasing the susceptibility to the SARS-CoV-2 virus. Nevertheless, these authors did not present any references to support the statement regarding chocolate’s unhealthy characteristics. It is interesting to note that only industrially processed foods were classified as UPF. Although processed foods, including packaging and additives, despite still needing improvement, are essential to food security control [12], similar foods prepared at home or in farms were not considered in NOVA classification [13].

## 3. Chocolate Types/Classifications

Primary cocoa processing allows the production of cocoa liquor (CL) (ground cocoa nibs), from which cocoa butter (CB) and cocoa powder (CP) that are both extracted from CL by pressing. These products can be mixed to sugar and milk products, frequently used at home, to obtain a solid or semiplastic food [14]. There are different standards of identity that establish required characteristics for cocoa and chocolate products. In this short review, only plain chocolate bars or tablets are considered. Readers interested in low sugar content chocolate processing are invited to read the very interesting review by Rad et al. [15].

Nowadays, there are many kinds of chocolate, all led by an array of gastronomical influences. Nevertheless, the classic, primary chocolate categories are dark, milk and white chocolates, which differ in their contents of cocoa solids, milk fat and CB [16]. Dark chocolate, also known as bittersweet or semisweet, usually contains at least 35% of total cocoa solids distributed between CL, CB and CP, and sugar and sometimes milk products. Milk chocolate contains a minimum of 15–25% of total cocoa solids and 12–14% total milk solids. White chocolate is a mixture of CB (at least 20%), milk products (minimum of 14% total milk solids) and sugar [17,18,19]. There are local and national standards that establish required minimum values and allowed ingredients for these chocolate kinds. However, manufacturers have considerable leeway for determining their formulations. Other chocolate categories were described by Merlino et al. [6] and Shafi et al. [16].

Recently, consumption of products with higher cocoa percentages has increased. For dark chocolate CL is within 45 to 80%, sugar runs between 20 and 55%, CB and lecithin are each from 0 to 5%, 0 to 0.5% and flavor is less than 0.5%, although a typically high cocoa solids recipe contains only CL (70%) and sugar (30%) [20]. Chocolate consists of a continuous fat phase where fine solid sugar, cocoa (and milk, if present) particles are suspended, forming a semisolid product [21]. The viscosity of this chocolate suspension depends on the coating of the solid particles with fat and an emulsifier can be added to aid this process [14]. Cocoa beans naturally contain phospholipids, which have surfactant action, and among these, phosphatidylcholine that is a major component of lecithin as an emulsifier [21,22]; however, in chocolate production, more lecithin can be added to ensure that the lyophobic sugar particles are coated with fat thereby significantly reducing its viscosity [20], but chocolate can be produced without it. Lecithin is a natural substance extracted from soybean and used in chocolate since the 1930s in amounts up to 0.5% [20,21,23]. Other emulsifiers can be employed like polyglycerol polyricinoleate (PGPR) in small amounts (up to 1%) depending on the desired viscosity [23]. This is very important because chocolate sensation arises from the way the chocolate is lubricated by fats and/or other ingredients in the chocolate itself or by saliva or a combination of both, controlling how solid cocoa particles are released, creating the tactile sensation [24].

The last 25 years have seen a growth of craft chocolate (also known as artisan, fine or “bean-to-bar”), frequently employed in gastronomy, that is produced by companies that source specialty cocoa beans and manufacture chocolate, controlling all the processing steps [25]. Craft chocolate consumers tend to prefer dark chocolate with cocoa percentages above 75%, which are perceived as high-quality products [26]. In addition to flavor and quality perception, higher cocoa percentage has been linked to greater content of free phenolic compounds and higher antioxidant capacity [27]. The main bioactive compounds found in chocolate are shown in Table 1 [28,29,30,31,32].

## 4. Chocolate Processing

**Figure 1:**
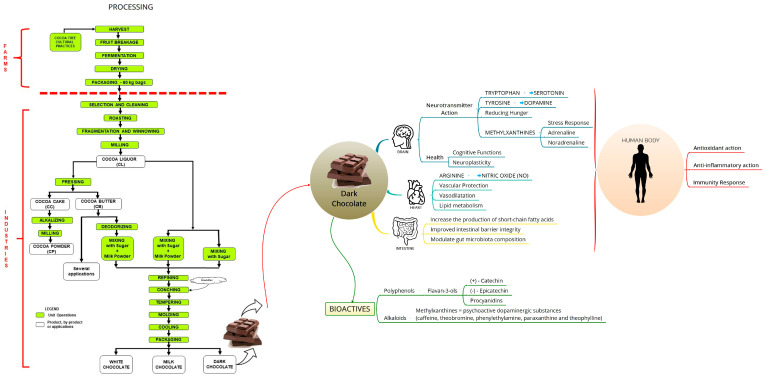
Chocolate processing, bioactive compounds and effects on human nutrition. Source: Own authorship. Icons from: chocolate (designed by Lebensmittelfotos from Pixabay; brain and heart (designed by Smashicons from Flaticon); intestine (designed by Kirill Kazachek from Flaticon); human body (designed by Freepik from Flaticon).

Chocolate is processed through a set of methods from cocoa beans into food, and the main steps from farm to consumer are summarized in Figure 1. Cocoa beans are harvested from the cocoa plant (*Theobroma cacao* L.), which is cultivated mainly within 20° north or south of the equator, and the fresh cocoa beans are preprocessed at the farms. There are three main cocoa groups, as follows:

(1) “Criollo” (approximately 2% of the world production) is considered as fine or flavor cocoa due to its genetic characteristics and typically mild flavor, low bitterness and aromatic quality, but it is susceptible to diseases and is found mainly in Ecuador and Venezuela;
(2) “Forastero” (85 to 90% of world production), found in the Amazonian region and dominant in Brazil, also known as common or bulk cocoa, considered astringent and bitter [18];
(3) “Trinitario” (roughly 13% of world production), which is a hybrid of the two other groups that also presents aromatic potential [33].

Cocoa preprocessing begins at farm level using cultural practices, harvesting, fruit opening, fermentation and drying. Fermentation is one of the main steps in chocolate processing and is performed in wooden boxes without addition of microorganisms [21,34]. Well-fermented cocoa beans are requisite for high-quality chocolate, while badly fermented cocoa can fail to provide specific aromatic compounds in chocolate. To ensure correct fermentation, it must last for seven consecutive days and then cocoa beans must be adequately dried [14,21]. Fermentation results from the successive action of several microorganisms and enzymatic actions, which are essential to produce the chocolate’s flavor and aroma precursors (peptides, free amino acids and reducing sugars) [34]. The hydrophobic amino acids are of particular importance in forming chocolate flavor precursors due to their bitter and astringent tastes [14].

First, an alcoholic fermentation occurs (anaerobic stages), where yeasts convert pulp sugars to ethanol followed by growth of acetic (AAB) and lactic bacteria (LAB) (aerobic stages) producing acetic and lactic acids, respectively, reducing pH from 7 to 5–5.5, which favors enzymatic action degrading the beans and increasing temperature to 45–50 °C [35]. During fermentation, the hydrolysis of sugars, organic acids and proteins and the oxidation of anthocyanins and polyphenols occurs [35]. Polyphenols are responsible for bean astringency and the initially purple color, and its content decreases due to oxidation by polyphenol oxidase and enzymatic darkening, which alters bean color to brown and reduces astringency [36].

After fermentation, bean moisture is between 50 and 60% and the beans are solar dried, where the enzymes produced during fermentation degrade compounds present within the beans, forming several different compounds and chocolate flavor precursors [7,14]. Drying proceeds until a moisture between 7 and 8% is reached then the beans are placed in 60 kg bags and transported to final industrial processing [14,21].

Industrial chocolate production has two main parts that can be performed at a single facility (bean-to-bar) or at separate facilities. The first part refers to bean processing and grinding to obtain CL and then CB and CP. Bean quality is assessed by evaluating moisture (below 8%) and a cut test is used to determine adequate fermentation, size, presence of off-flavors and number of defects (flat, moldy, infestations, etc.) [21]. According to chocolate type and cocoa percentage in the product, different bean characteristics are desirable. Chocolatiers who make craft chocolate source beans regarding flavor and high quality (i.e., well fermented, no defects and no off-flavors) [21,26]. The selected beans undergo cleaning to remove sand, stones, twigs and other impurities that may come from the farm. Beans are then roasted, broken and winnowed to remove shells and obtain nibs (ground kernels) of relatively uniform size. These operations can be performed in different order and under different conditions according to chocolate type and manufacturer preference, but all are necessary to obtain CL. Roasting is conducted in rotary drum roasters or continuous roasters where the cocoa beans are heated from ambient temperature to temperatures between 110 and 160 °C, according to cocoa bean types [37]. Roasting is a complex operation but essential in developing chocolate flavor using the Maillard reaction, Strecker degradation, oxidation of lipids and polyphenols, and reducing moisture below 2% [16,38]. During this operation, volatile and nonvolatile chocolate flavor compounds are formed, as well as the characteristic brown color [14,21,37]. Roasting is the only unit operation in bean-to-bar processing that is carried out at temperatures above 100 °C. To increase energy efficiency, the heat from the roasting medium can be recovered and used in conching, for example.

The beans can be sterilized to reduce microbial counts (especially if roasting temperatures and times are low) and/or alkalized to enhance flavor, color and dispersibility in water. The nibs are then ground to produce CL, and the grinding process is multistage to reduce particle sizes from roughly 0.5 cm to below 40 µm, otherwise they would be detected in the mouth as “sandiness” [39], although a value between 20 to 25 µm is more suitable [40]. This operation can be performed employing different equipment (knife mills, disc mills, ball mills, refiners, among others). Small particle sizes ensure coating of particles with fat and define chocolate viscosity, texture and mouth feel [14,21]. Meanwhile CB, produced from CL by pressing, is deodorized by vacuum steam distilling, while the press cake is pulverized to make CP [14].

These ingredients obtained from cocoa beans then go to a second stage, whose schematics can be viewed in Petrus et al. [13]. This phase is related to the final form of chocolate making, comprising the following steps:

(1) Mixing cocoa products with sugar, milk, or other ingredients according to chocolate type and formulation and the ensuing processing steps for obtaining the solid chocolate bar;
(2) After mixing, the liquid chocolate paste is refined to further reduce particle sizes, ensuring flowability and adequate sensory properties. During refining, temperatures can reach 60 °C because of viscous dissipation, but cooling is applied to reduce the temperatures so that adequate viscosity is maintained throughout the process [14];
(3) The chocolate is then conched, which is a stirring operation at temperatures above 50 °C for a few hours to remove undesirable volatile compounds to reduce acidity and further develop flavor as well as viscosity and texture [41]. Conching is crucial in chocolate processing for the development of sensory and quality characteristics [42];
(4) The chocolate undergoes tempering to promote adequate fat crystallization in cocoa butter’s most thermodynamically stable form [16,39]. Fat crystallization and its control are critical in defining chocolate quality [43]. During tempering, chocolate is first heated above 50 °C to ensure that all the crystals are melted, then cooled to approximately 27 °C, where both stable and unstable crystals are formed, then reheated to 30–32 °C, so that the unstable crystals melt and only the stable crystals remain. If tempering is correctly performed, the proportion of β_V_ crystals obtained provides the desired sensory quality in the chocolate, so that the product presents the desirable brightness, the proper melting point, hardness (good snap) and shelf-life (avoiding fat bloom, for instance) [14,44];
(5) Tempered chocolate is ready for molding into various forms (bars, shells) or for coating and enrobing chocolate products. The chocolate products are cooled to remove specific heat (at about 12–15 °C for 5 min), then further cooled to remove latent heat (about 7–10 °C for 10–20 min) and then slightly heated to a temperature above the dew point of the packing area to avoid moisture condensation on the chocolate surface. Chocolate can then be demolded and packed [14].

Chocolate quality is complex and is influenced by the combination of all these processes, which must be tailored to obtain the desired results [21]. Chocolate must be stored under controlled conditions (<22 °C) to avoid fat blooming, a common consequence of temperature fluctuations (heating/cooling) [45].

It is evident, therefore, that chocolate processing involves biotechnological (at the farm) and physical (in industry) phenomena, and that there are no chemical processes in chocolate production. Chemical changes that occur during chocolate processing are a consequence of these other processes [13]. More detailed description on chocolate processing has been provided by Shafi et al. [16], information on processing effect on the component changes of chocolate during bean-to-bar processing can be found in Kitani et al. [25], while the effect of processing on bioactive compounds was reviewed by Goya et al. [46] and the most important chemical reactions that occur with proteins, carbohydrates, lipids, and polyphenols during chocolate processing were reviewed by Barišić et al. [38].

## 5. Chocolate’s Nutrients: Health and Risks

Overall, cocoa contains a significant amount of fat (25.6%) followed by protein (20.4%), while also serving as a valuable source of minerals such as potassium, phosphorus, copper, iron, zinc, and magnesium [1]. Clearly, the composition of chocolate depends on its formulation, and it is rich in fat due to the addition of cocoa butter into the formulations.

Notwithstanding, there are controversies, including some myths, about chocolate consumption [16]. For example, there is a myth that chocolate is very high in caffeine. Dark chocolate can be high in caffeine, but not as high as many people think (1 bar can have 10 times less caffeine than a “grande” brewed coffee) [16]. Moreover, as described previously, chocolate has been considered an UPF [11], and then, an unhealthy food [10], which means that its consumption must be avoided. UPF definition does not refer to unit operations required for food processing, but to ingredient numbers and, mainly, to additives not commonly employed in culinary preparations [47]. As previously described, the number of ingredients in dark chocolate cannot be considered high, no unknown additives are used during its processing, and there is no chemical synthesis or extraction of substances from whole foods, so the advice to avoid dark chocolate consumption can overlook its considerable health benefits if it is consumed moderately, avoiding problems that might be linked to its high fat and sugar contents.

Cocoa is a crop with one of the highest contents of bioactive compounds in the plant kingdom. Cocoa beans contain more than 300 identifiable chemical compounds. Several of these compounds have remarkable qualities, highlighted by their antioxidant and anti-inflammatory action that may have beneficial effects against the development of degenerative and neurodegenerative diseases. They can also act to modulate lipid metabolism and composition of the intestinal microbiota [48,49]. The main bioactive compounds responsible for the health benefits of cocoa and cocoa-based products are shown in Figure 1, such as free bioactive amines and amino acids (tryptophan, tyrosine, arginine), which affect the human body and play an important role in human health [50]. The potential health benefits of consuming dark chocolate or cocoa bioactive compounds can be seen in the studies presented in Table 2, which emphasizes the consideration of the trial designs.

Dark chocolate contains flavonoids, including catechins, epicatechin, and procyanidins and methylxanthines (caffeine, theobromine, phenylethylamine, paraxanthine and theophylline) from cocoa beans, responsible for the higher intensity of bitterness, cocoa flavor, acid taste and astringency in the product [32]. Moreover, methylxanthines are psychoactive dopaminergic substances responsible for its pleasurable effects and influence on plasmatic levels of stress hormones such as cortisol and catecholamines (both adrenaline and noradrenaline) [30,31]. All these compounds can directly or indirectly affect the cardiovascular system by multiple mechanisms. Cocoa polyphenols modulate inflammatory markers and induce nitric oxide release by activating endothelial nitric oxide synthase which, in turn, accounts for vasodilation and cardioprotective effects [30,57]. A randomized, double-blind, placebo-controlled crossover trial using epicatechin, a cocoa flavan-3-ol, found that the gene expression involved in inflammation was reduced, as well as PPAR signaling and adipogenesis in immune cells, indicating that cocoa consumption might have an impact on immune cells and potentially offer protective cardiometabolic effects [62].

Chocolate consumption at <100 g/week may be associated with reduction in cardiovascular disease risks, including stroke, heart failure, myocardial infarction and coronary heart disease [55]. Moreover, dark chocolate can exert antioxidant activity and improve endothelial function via downregulation of oxidative stress generated by nicotinamide adenine dinucleotide phosphate oxidase isoform 2 (NOX-2), which is considered a pathogenic mechanism that determines fibrosis and disease progression in nonalcoholic steatohepatitis in humans [69,70]. Nevertheless, the works of systematic reviews and meta-data analyses about chocolate consumption associated with effects on health were reviewed and it has been concluded that there is weak evidence for the effects of chocolate consumption on health outcomes in diseases of diabetic or cardiovascular nature [71]. Moreover, according to Morze et al. [72], evidence for the association between chocolate intake and risk of chronic diseases is inconclusive.

Milk and white chocolate’s effect can be more convoluted, however, because sucrose and lipids may transiently and negatively influence endothelial function, partly through insulin signaling and nitric oxide bioavailability [73], although chocolate presents a low glycemic index. The ingestion of a very high amount of chocolate (100 g) in a narrow time window (1 h) in the morning can help to burn body fat and lower glucose levels in postmenopausal women, as well as reduce hunger sensation, the desire for sweets and ad libitum energy intake by ~300 kcal during the day; however, this did not fully compensate for the extra energy contribution of chocolate (542 kcal/day) [61]. A systematic review on whether dark chocolate supplementation has a favorable effect on body weight and body mass index (BMI), revealed a significant reduction in body weight and BMI with chocolate consumption ≥ 30 g/day during between 4 and 8 weeks [71].

Research on eating behaviors among overweight, obese and normal body weight individuals shows that greater emotional eating results in greater desire for chocolate consumption; therefore, an approach considering eating habits and lifestyle suggests that the best way to consume chocolate is to be attentive to the amount, type and frequency of chocolate consumed in the daily diet. Also, chocolate consumption can modify the intestinal biota and increase the production of short-chain fatty acids (such as acetate, propionate, isobutyrate, isovalerate and valerate); this can significantly reduce the sensation of hunger [61]. Additionally, it is known that chocolate consumption can (positively or negatively) modify consumer mental states by impacting their emotions and cognitive and sensorial responses [74].

Moreover, chocolate can be processed as a functional food by incorporating probiotics or bioactive compounds that enhance its healthy characteristics. In this regard, semisweet chocolates incorporating probiotics Lactobacillus acidophilus LA3 and Bifidobacterium animalis subsp. lactis BLC1 in an amount of 108 cfu/g were developed by Silva et al. [75]. These authors developed a new functional food by combining the health benefits of probiotics with those of chocolate’s phenolic compounds. Islam et al. [76] determined that the milk chocolate matrix can potentially be used as a carrier of probiotics (L. acidophilus LDMB-01) with optimum probiotic viability and unaltered physicochemical properties. Meanwhile, a 70% cocoa chocolate containing ursolic and oleanolic acids isolated from Mansoa hirsuta DC (a plant from the Brazilian semiarid region rich in triterpene acids), that possesses anticholesterolemic, hypoglycemic, antihepatotoxic, antioxidant, anti-inflammatory, antifungal and antibiotic activities was developed by Milagres et al. [77]. Powdered chocolate can be used as a probiotic encapsulant, ensuring viability during chocolate processing and storage and protection from digestion in the gastrointestinal tract [67,76]. Microbiome modulation is a strategy in evolution to improve human health that is part of a comprehensive and holistic wellness lifestyle approach and chocolate can be an adjunct in this regard [59].

## 6. Conclusions

Chocolate is a popular confectionery product whose consumption has been increasing over the years, particularly that of dark chocolate. Chocolate is produced with varying amounts of CL, CB and CP, extracted from fermented and dried cocoa beans, according to chocolate type. Milk products, sugar and an emulsifier can be also added in its formulation depending on chocolate type.

Chocolate manufacture involves biotechnological and physical processes, in farms and industries, respectively. Industrially, chocolate processing involves a relatively low number of unit operations (mainly, mechanical and thermal operations), does not involve chemical synthesis or extraction of substances from whole foods and no unknown additives are used, unlike UPFs. Chocolate can even be produced without emulsifier.

It is true that chocolate is an energy-dense food, and because of that, its consumption must be moderate, preferably as part of a balanced diet. Moreover, it must be noted that it is also an indulgent food, not a medicine. Nevertheless, chocolate is rich in bioactive compounds associated to health benefits regarding cardiovascular risk, improving cognitive function, mitigating oxidative and inflammatory burden in degenerative diseases, maintenance of diversity in gut microbiota, weight loss and other benefits and thus should not be considered an unhealthy food. Given this, to classify it as a UPF without considering its low processing level (with no chemical synthesis or extraction of substances), composition (few ingredients) and potential health benefits when consumed moderately is arbitrary, and it should not be considered as a UPF.

## Figures and Tables

**Table 1:** Main bioactive compounds present in chocolate *.

Compound	Molecular Formula	Chemical Structure	References
Polyphenols			
(+)-catechin	C_15_H_14_O_6_	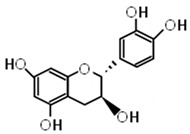	[29,30,32]
(-)-epicatechin	C_15_H_14_O_6_	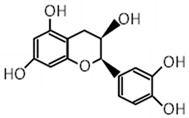	[29,30,32]
Procyanidin A2	C_30_H_24_O_12_	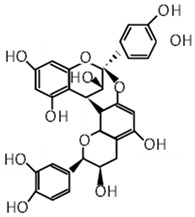	[29,30]
Alkaloids			
Theobromine	C_7_H_8_N_4_O_2_	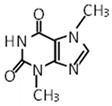	[30,31,32]
Caffeine	C_8_H_10_N_4_O_2_	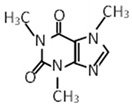	[30,31,32]
Phenylethylamine	C_8_H_11_N	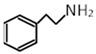	[30]
Paraxanthine	C_7_H_8_N_4_O_2_	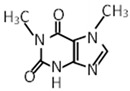	[30]
Theophylline	C_7_H_8_N_4_O_2_	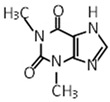	[30,31]

* Chemical structures were obtained from ChemSpider [28].

**Table 2:** Studies presenting benefits of dark chocolate consumption and/or of bioactive compounds present in chocolate.

Compound	Trial Design	Effect	References
Cardiovascular health			
Dark chocolate	- Systematic review, meta-analysis, and dose–response analysis of randomized controlled trials. - Meta-analysis of prospective studies. - Overweight (BMI 25–32 kg/m^2^), middle-aged male subjects between 45 and 70 years old; 70 g of chocolate daily in two 4-week periods. - A randomized trial. - In vitro and in vivo study of seed-derived peptides (*Theobroma cacao* L.).	- Improved flow-mediated dilatation, reduced arterial stiffness, especially in men. - Reduces the risk of cardiovascular disease, including stroke, heart failure, myocardial infarction and coronary heart disease. - Improves leukocyte adhesion factors and vascular function in overweight men. - OxLDL concentration significant decrease of the concentration of OxLDL and total cholesterol, triglycerides, and LDL-cholesterol. Increase in plasma HDL cholesterol. - Reduces blood pressure by blocking angiotensin-converting enzyme.	[51,52,53,54,55]
Polyphenols	- Review. - In vitro study.	- Modulates inflammatory markers and induces nitric oxide release through activation of endothelial nitric oxide synthase, which is responsible for vasodilation and cardioprotective effects. In addition: antioxidant properties, antihypertensive effects, modulation of eicosanoids. - In vitro study interferes with oxysterol-mediated inflammation.	[30,56,57,58]
Flavonoids	- Review. - In vivo study. - Male participants (those under 18 years old and over 45 years old were excluded).	- Flavonoids exhibit cardio- and neuroprotective effects, as demonstrated with improvements in flow-mediated dilation response, reductions in blood pressure and increases in cerebral blood flow. - Preventive effect of cocoa flavanols against glucotoxicity-induced vascular inflammation in the arteria of diabetic rats and on the inflammatory process in the endothelial cells. - Flavanols are effective at counteracting mental stress-induced endothelial dysfunction and improving peripheral blood flow during stress.	[59,60,61]
Epicatechin	A randomized double-blind, placebo-controlled crossover trial.	- Epicatechin, a cocoa flavan-3-ol, reduces gene expression involved in inflammation, PPAR signaling and adipogenesis in immune cells; affects immune cells and exerts cardiometabolic protective effects.	[62]
Catechins	Review	- A key mediator in cardiovascular health via mechanisms of blood pressure reduction, flow-mediated vasodilation and atherosclerosis attenuation.	[63]
Cognitive function			
Flavonoids	- Systematic review and analysis of methodological aspects.	- Positive effects on cognitive functions with epicatechin consumption of 50 mg/day or more using cocoa as a vehicle in young adults.	[48,64]
	- Review.	- Beneficial effect of cocoa flavanols on cognitive function and neuroplasticity and indications that such benefits are possible in early adulthood.	
Antioxidant action			
Epicatechin	In vitro study: monomeric flavanol epicatechin in human endothelial cell culture subjected to an oxidative challenge.	- Flavonoids protect human endothelial cells against an oxidative insult by modulating oxygen radical generation and antioxidant enzyme and nonenzymic defenses.	[65]
Probiotic action			
Dark chocolate	- A randomized controlled trial. - In vitro digestion model. - In vivo study.	- Increases the production of short-chain fatty acids (such as acetate, propionate, isobutyrate, isovalerate and valerate) and reduces hunger. - The dark chocolate offered a good protection to probiotics during simulated gastrointestinal transit.	[61,66,67,68]
		- Modulates gut microbiota composition, improved intestinal barrier integrity and intestinal inflammation, increases total levels of short-chain fatty acids, improves intestinal health in diabetic rats.	

## Data Availability

The data used to support the findings of this study can be made available by the corresponding author upon request.
